# The Interaction and Implication of Stress-Induced Hyperglycemia and Cytokine Release Following Traumatic Injury: A Structured Scoping Review

**DOI:** 10.3390/diagnostics14232649

**Published:** 2024-11-24

**Authors:** Ibrahim Al-Hassani, Naushad Ahmad Khan, Eman Elmenyar, Ammar Al-Hassani, Sandro Rizoli, Hassan Al-Thani, Ayman El-Menyar

**Affiliations:** 1 Weill Cornell Medical College, Doha P.O. Box 24144, Qatar; iaa4004@qatar-med.cornell.edu; 2 Department of Surgery, Clinical Research, Trauma & Vascular Surgery, Hamad Medical Corporation, Doha P.O. Box 3050, Qatar; naushadkhan82@gmail.com; 3 Faculty of Medicine, Bahcesehir University, Istanbul 34734, Türkiye; eelmenyar@gmail.com; 4 Department of Surgery, Trauma Surgery, Hamad Medical Corporation, Doha P.O. Box 3050, Qatar; ammar_alhassani@yahoo.com (A.A.-H.); srizoli@hamad.qa (S.R.); 5 Department of Surgery, Trauma and Vascular Surgery, Hamad Medical Corporation, Doha P.O. Box 3050, Qatar; althanih@hotmail.com; 6 Department of Clinical Medicine, Weill Cornell Medical College, Doha P.O. Box 24144, Qatar

**Keywords:** trauma, hyperglycemia, stress-induced, cytokines, mortality, structured scoping review

## Abstract

Introduction: This is a structured scoping review to assess whether there is a relationship between stress-induced hyperglycemia (SIH), cytokine interactions, and mortality in trauma patients in comparison to non-diabetic normoglycemia [NDN], diabetic normoglycemia [DN], and diabetic hyperglycemia [DH]. Methods: We conducted a literature search of MEDLINE (PubMed) databases from 2000 to 2022 using a search strategy to identify observational studies. Initially, 2879 articles were retrieved. Of these, 2869 were excluded due to insufficient variables, and non-trauma focuses. Results: Nine studies on the interaction between SIH and proinflammatory cytokines were analyzed. SIH was associated with the highest mortality rate (21.3%), followed by DH (5.4%), DN (2.8%), and NDN (2.3%) (*p* < 0.001). Furthermore, SIH patients exhibited an 11.28-fold higher likelihood of mortality compared to NDN patients (95% CI [9.13–13.93]; *p* < 0.001) and a 4.72-fold higher likelihood compared to DH patients (OR 4.72; 95% CI [3.55–6.27]; *p* < 0.001). Conclusions: SIH patients had elevated IL-6 concentrations relative to NDN, DN, and DH patients. SIH is linked to higher mortality in trauma, with greater odds than NDN. However, the robustness of this association is still being determined due to statistical and clinical variability. Uncertainties about injury severity and IL-6 level similarities between SIH and DH patients require further investigation.

## 1. Introduction

Traumatic injuries are a leading global cause of death [1,2]. Acute insults, in the form of trauma and stress, trigger a sequence of inflammatory responses (inflammatory cascade) that can lead to hyperglycemia. This phenomenon disrupts the metabolic equilibrium between glucose production and insulin secretion [3]. Hyperglycemia induced by acute injury, known as stress-induced hyperglycemia (SIH), is the transient elevation of blood glucose levels triggered by acute stress and involves interactions between the immune, endocrine, and nervous systems. This leads to hypermetabolic response [4,5], a state of temporary insulin resistance in the peripheral tissues and concomitant relative insulin deficiency [5,6,7], increased stress hormones, and inflammatory responses by high circulating levels of cytokines [8], and increased oxidative stress and immune dysfunction [9]. The incidence of SIH varies with the type of trauma. It was reported in 4–5% [10] of all trauma cases and in 8% [11] of patients with moderate to severe isolated traumatic brain injuries (TBIs). Notably, the highest prevalence is documented in patients with hip fractures, at around 49% [12].

Recent studies indicate that SIH is associated with more severe illness and higher short-term mortality rates [13,14,15,16]. However, this condition typically subsides after the recovery from acute illness, so it has not been traditionally considered detrimental to long-term health consequences [3]. Nonetheless, the acute stress might expose pre-existing latent issues such as insulin resistance, characterized by impaired pancreatic β-cell function, which affects insulin production. These underlying conditions may not be evident under normal circumstances but become apparent during the stress of a critical illness [17,18]. This could result in an elevation in glucose levels, adversely affecting the body, including organ system failure [19,20].

SIH has become a widely researched topic, encompassing its biochemical origins, natural course, management strategies, and impact on patient outcomes. Traditionally, SIH was viewed as a protective mechanism during acute injury [21]. However, research over the past two decades has revealed a positive [22,23,24] as well as an unfavorable correlation between SIH in trauma patients and outcomes [11,25,26,27,28,29]. Recent research has linked SIH to significantly higher mortality and morbidity in critically ill trauma patients rather than diabetic hyperglycemia (DH) [30,31,32]. Notably, these patients have a three-fold higher likelihood of mortality compared to those who maintain non-diabetic normoglycemia (NDN) [11]. This distinction highlights SIH as a physiological process with potential ramifications for developing targeted management strategies in trauma patients [3,19].

While some studies have found a clear association between SIH and mortality in trauma patients, others have reported discrepancies [10,11,29,30,32,33,34]. Consequently, SIH remains one of the controversial areas of investigation and scrutiny within trauma research settings. This controversy has prompted further exploration into the role of proinflammatory cytokines associated with trauma mortality [34,35]. Proinflammatory cytokines, such as interleukin-6 [1] (IL-6, IL-1) and tumor necrosis factor-α (TNF-α) play significant roles in the development of insulin resistance and hyperglycemia in trauma patients [36,37,38,39,40,41]. The stress response is associated with an increased secretion of these proinflammatory cytokines from immune cells and other tissues [42]. This immunological response stimulates the release of counter-regulatory hormones, which increase glucose synthesis in the liver, decrease glucose absorption in peripheral tissues, and cause insulin resistance in skeletal muscle and liver cells [43], leading to SIH [44,45]. Understanding the interplay between SIH and cytokines is crucial, as traumatic injuries are a significant cause of death, especially in younger individuals [46,47].

The preceding literature has not yet established whether SIH and cytokine-mediated inflammation are concurrent phenomena with comparable sequelae or if SIH and inflammation in diabetic patients constitute a distinct clinical entity from non-diabetic patients, leading to unique clinical responses and outcomes. Therefore, this review explores the differential impact of SIH on trauma outcomes, specifically mortality, among different glycemic presentations (NDN, diabetic normoglycemia [DN], and DH). Furthermore, we also aimed to explore the interaction between SIH and proinflammatory cytokines in trauma patients and their association with SIH and the outcome.

### 1.1. Pathophysiology and Mechanism of SIH

The stress response is orchestrated primarily by the interplay of several physiological systems, namely the hypothalamic–pituitary–adrenal (HPA) axis, the sympathetic nervous system (SNS), and the sympathoadrenal system (SAS) [48]. Together, these systems work synergistically to mobilize resources and adapt the body’s functions to cope with stressors.

Fundamentally, traumatic injuries trigger an initial physiological response that increases the availability of metabolic substrates for energy generation. These substrates include amino acids, free fatty acids, and, most notably, glucose [7,45]. In the brain, the hypothalamus and brainstem function as the glucose regulatory center, controlling blood sugar levels in response to stress reactions. The catecholamine neuronal system’s ventrolateral medulla (VLM) primarily regulates stress control [49]. The hypothalamic paraventricular nucleus (PVN) sends signals to the catecholamine neurons of the ventrolateral medulla (VLM) that control SIH [50].

Notably, stress leads to the activation of the HPA, which triggers an acute metabolic response. The hypothalamus releases corticotropin-releasing hormone (CRH), which stimulates the SANS and SAS and influences the pituitary gland. This leads to adrenocorticotropic hormone (ACTH) release into the bloodstream [48]. ACTH travels to the adrenal glands, prompting them to secrete cortisol (also known as the stress hormone) via the CRF-ACTH axis, which, in turn, raises blood sugar levels by lowering peripheral glucose absorption and promoting hepatic gluconeogenesis [51]. This causes cortisol to be secreted by the corticotropin-releasing factor—adrenocorticotropic hormone.

Stress also activates the SANS, which is responsible for the immediate “fight-or-flight” response to stress. When activated, it triggers the release of adrenaline (epinephrine) and noradrenaline (norepinephrine) from the adrenal medulla into the bloodstream by activating catecholaminergic neurons in the brainstem and efferent neurons in the spinal cord. Epinephrine and norepinephrine, hormones associated with adrenergic activity, directly inhibit insulin secretion [52]. Furthermore, epinephrine reduces hepatic glycogen synthesis while simultaneously promoting glycogenolysis and hepatic gluconeogenesis. Moreover, it alters post-receptor signaling pathways, causing an insulin-resistant effect on skeletal muscles [3,19,53].

**Figure 1:**
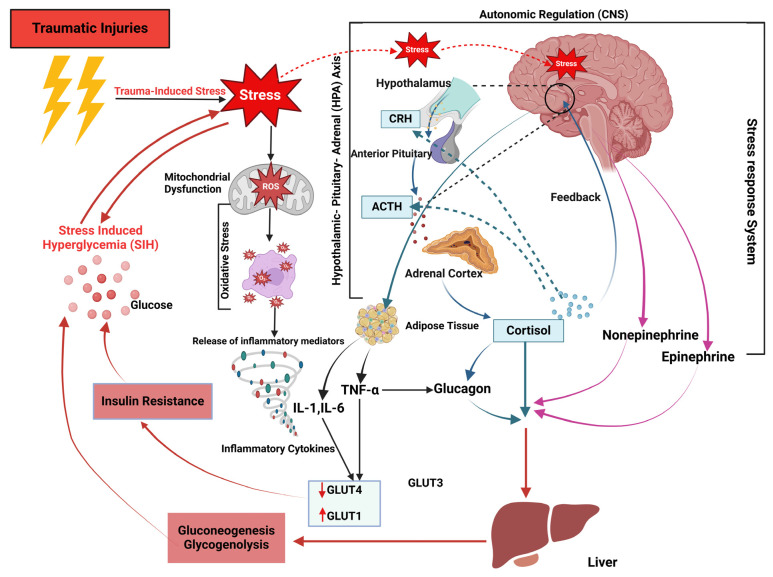
Mechanisms of the immune–neuroendocrine axis and stress-induced hyperglycemia post-trauma. Hepatic gluconeogenesis, glycogenolysis, and insulin resistance primarily cause the generation of stress-induced hyperglycemia. Trauma-induced stress activates the sympathoadrenal system and the HPA axis. Stress causes the adrenal medulla to release catecholamines through the PVN-VLM-IML pathway. This, in turn, causes the adrenal cortex to make cortisol through the HPA axis. Cortisol, along with IL-6, IL-1, and TNF-α from surrounding tissues, can stimulate glucagon. Glycemic hormones such as catecholamines, cortisol, and glucagon act on the liver to promote hepatic gluconeogenesis and glycogenolysis. Hyperglycemia exacerbates stress response and increases the release of proinflammatory factors, inciting an inflammatory response. Figure 1 was created using Biorender.com.

Glucagon, catecholamines, and cortisol work together to influence glucose metabolism. In typical circumstances, increased insulin levels in the bloodstream promote glucose storage with the help of the insulin-responsive glucose transporter GLUT-4, primarily in muscle and fat tissues [3,20]. In cases of severe traumatic injury, there is a decrease in the ability of insulin to facilitate the absorption of glucose. At the same time, there is an increase in the expression of glucose transporters GLUT-1 and GLUT-3 in different tissues [54]. This inhibits glucose storage through GLUT-4 and worsens glucose levels in the peripheral circulation [3,21]. The HPA axis and the sympathetic-adrenal system (SAS) stimulate the overproduction of proinflammatory cytokines such as IL-6, IL-1, and TNF-α. These systems collectively and synergistically contribute to the development of stress-induced hyperglycemia in trauma patients without diabetes [3] (Figure 1).

### 1.2. Role of Counter-Regulatory Hormones and Proinflammatory Cytokines

Complex interactions between neurohormonal and hepatic autoregulatory mechanisms typically regulate blood glucose levels. These regulatory processes involve the CNS and hormonal signals modulating hepatic functions [55]. Specifically, the liver plays a crucial role in glucose metabolism through two primary processes: glycogenolysis and gluconeogenesis. These mechanisms work in tandem to ensure stable blood glucose concentrations and respond dynamically to metabolic demands and stress conditions [56].

Counter-regulatory hormones exert their effects by modulating gene expression and inhibiting specific processes to stimulate the production of new glucose in the liver (gluconeogenesis) and reduce the effectiveness of insulin, resulting in hyperglycemia. Cortisol, epinephrine, and norepinephrine stimulate the production of the enzyme phosphoenolpyruvate carboxykinase (PEPCK), which plays a critical role in gluconeogenesis [57]. This metabolic pathway converts non-carbohydrate substrates, such as lactate, alanine, and glycine, into glucose that subsequently leads to hyperglycemia [3,58,59].

The activation of the HPA axis during the acute phase of trauma leads to higher levels of cortisol and corticotropin, emphasizing glucocorticoid synthesis [60,61]. Concurrently, the activation of the sympathetic nervous system raises levels of counter-regulatory hormones such as adrenaline, norepinephrine, glucagon, and growth hormone. Catecholamines (ChAs), such as epinephrine and norepinephrine, can cause insulin resistance by suppressing the activity of insulin receptor substrate (IRS)-1. Moreover, ChAs prevent insulin-mediated glucose uptake in cells by inhibiting insulin binding to its receptors and blocking tyrosine kinase activity [20,62]. Similarly, growth hormones have the potential to induce insulin resistance by diminishing insulin receptors and compromising tyrosine kinase activity [63]. As a result, insulin loses its ability to effectively regulate blood glucose levels, leading to a condition known as insulin resistance. The elevated blood sugar levels caused by this can stimulate the transcription factor nuclear factor-kappa B (NF-κB) to enhance the production of IL-1, IL-6, and TNF-α [3,53].

**Figure 2:**
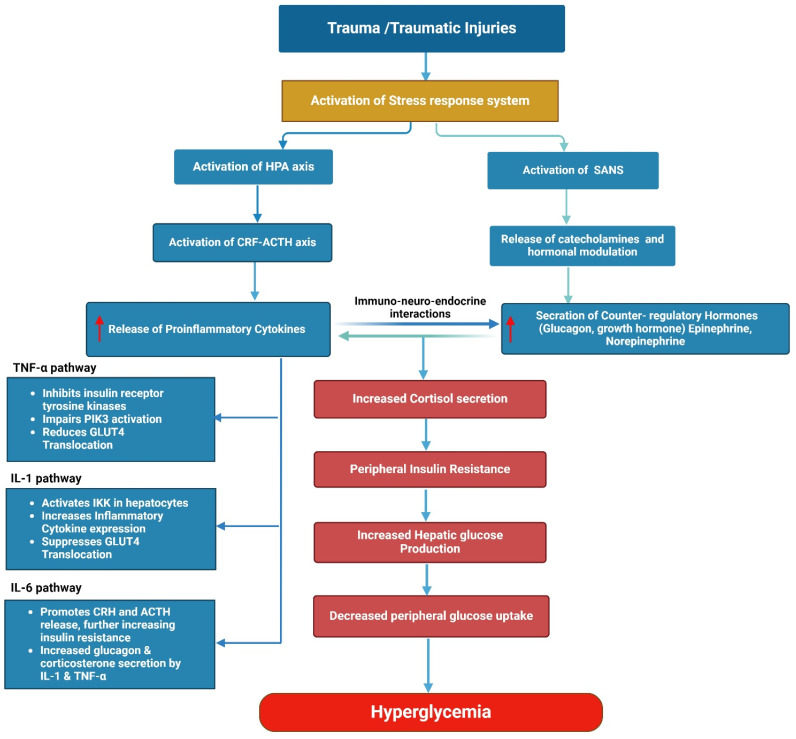
The role of proinflammatory cytokines and counter-regulatory hormones in SIH and its adverse outcomes.

Proinflammatory cytokines induce gluconeogenesis and insulin resistance by interacting with counter-regulatory hormones (Figure 2). For example, TNF-α stimulates the release of counter-regulatory hormones, which in turn enhances the processes of gluconeogenesis and glycogenolysis. Similarly, IL-6 stimulates the occurrence of insulin resistance and high blood sugar levels by triggering the release of CRH and ACTH. Similarly, the action of IL-1 stimulates the release of glucagon and corticosterone, promoting glucose production and ultimately resulting in hyperglycemia [3,53]. However, cytokines can affect the process of gluconeogenesis and insulin resistance without having an impact on counter-regulatory hormones. TNF-α, ChAs, and growth hormones induce insulin resistance by inhibiting tyrosine kinases and the tyrosine phosphorylation of IRS-1 [64,65]. In addition, elevated IL-6 levels induce insulin resistance and hyperglycemia by mobilizing glucose from hepatic glycogen stores. In these mechanisms, the role of cytokines inducing gluconeogenesis and insulin resistance through the elevation of counter-regulatory hormones prompts the question of whether the adverse effects observed in SIH patients are due to the initial stressor or are driven by cytokines. This question was the primary rationale for including cytokines in the current review.

### 1.3. Adverse Effects of SIH Following Traumatic Injury

Glucose is the brain’s primary energy source, and its absorption into cells is facilitated by plasma membrane glucose transporters (GLUTs), which enable glucose to move across the lipid cell membrane. Among the different isoforms identified in humans, GLUT-1, GLUT-3, and GLUT-4 are responsible for glucose uptake in various organs. Insulin enhances GLUT-4-mediated glucose transport by promoting its translocation to the cell membrane [53]. SIH leads to glucose transporter (GLUT) overexpression, causing glucose overload and glucotoxicity (decrease in insulin secretion and increase in insulin resistance) in cells expressing these transporters [20,66]. This increases reactive oxygen species (ROS) production (i.e., peroxynitrite and superoxide) and causes oxidative stress (imbalance between ROS and antioxidants) through dysfunction that may occur alongside altered energy metabolism, apoptosis, cellular and organ system failure, inflammatory cascades, and endothelial injury [20,60].

Moreover, acute insulin resistance results in decreased glucose uptake due to impaired post-receptor insulin signaling and the downregulation of GLUT-4. Proinflammatory cytokines such as TNF-α, IL-1, and IL-6 can interfere with post-receptor insulin signaling [3]. Following trauma-induced SIH, the overexpression of GLUT-1 and downregulation of GLUT-4 may facilitate the redistribution of glucose from peripheral tissues and the neurological system toward the immune system and immune cells. Additionally, TNF-α may inhibit the expression of GLUT-4 messenger RNA, further reducing glucose transport into cells [67]. When the sympathetic nervous system is activated, it leads to the breakdown of adipocytes, releasing increased levels of free fatty acids [68]. These excess free fatty acids can block insulin signaling pathways and inhibit glycogen synthase, a key enzyme in glucose storage, thereby reducing glucose absorption [69]. Due to the metabolic interactions, the homeostasis of trauma patients is disrupted, which may explain the higher mortality observed in SIH patients.

Moreover, adipose tissue produces high levels of proinflammatory cytokines, including monocyte chemotactic protein-1 (MCP-1), IL-6, and IL-1, which exacerbate insulin resistance [70]. TNF-α also contributes to elevated blood glucose levels by stimulating glucagon production, a hormone that increases glucose release from the liver [71]. These mechanisms and adverse effects suggest an interlinked mechanism between cytokines and SIH.

Notably, proinflammatory cytokines must be present in high concentrations for the upregulation of GLUTs and glucose overload without insulin influence [53,60]. However, for the proinflammatory cytokine concentrations to be elevated, glucose must be present to activate the inhibitor κB kinase (IKK) and nuclear factor-kappa B (NF-κB) [72,73]. Glucose increases transcription factors such as nuclear factor-kappa B (NF-κB) in the nucleus [74]. Normally, NF-κB in the cytoplasm is bound to the inhibitory protein IκB, keeping it inactive. Inflammatory stimuli cause the phosphorylation and degradation of IκB, allowing the active NF-κB subunits (p50 and p65) to move to the nucleus [20,74]. This triggers the production of inflammatory proteins like TNF-α, IL-1, IL-6, and MCP-1 [75]. Hyperglycemia stimulates the NF-κB subunit p65 promoter, enhancing gene expression that produces these inflammatory factors. Additionally, NF-κB may activate ICAM-1, VCAM-1, and E-selectin, contributing to the inflammatory response [76]. This suggests an interdependence between SIH and cytokines that could explain why they are both associated with more significant mortality in trauma, as it may not be the independent action of one but the combined action of both that causes this association.

## 2. Materials and Methods

Search strategy and data sources: The current systematic review follows the guidelines outlined by the Preferred Reporting Items for Systematic Reviews and Meta-Analyses (PRISMA) and specific protocols for reporting and conducting systematic reviews of incidence and prevalence studies. A comprehensive literature search was conducted for relevant articles published between January 2000 and December 2022. We used MeSH terms and free-text keywords to identify relevant articles. To investigate the association between SIH and mortality, we searched the PubMed (Medline) database using the terms “stress-induced hyperglycemia”, “mortality”, and “trauma”. For the cytokine review, the search terms included “cytokines”, “IL-6”, “IL-1”, “TNF-α”, “mortality”, and “trauma”. Additionally, we examined the reference lists of published reviews to identify any further pertinent studies. Detailed information regarding the search strategy is provided below.

### 2.1. Inclusion and Exclusion Criteria

Studies meeting the following criteria were included in this systematic review analysis:

1. Age and gender: Studies involving adults aged 16 and older and both genders were included. This ensures a focus on a population with stable and comparable physiological and medical responses, making the data more reliable. Studies involving pediatrics and pregnant women were excluded.
2. Study type: Only cohort (prospective or retrospective) studies conducted in trauma patients were included. Prospective studies observe real-time outcomes from a specific starting point, while retrospective studies analyze records to evaluate historical outcomes. Both types offer valuable insights into trends and outcomes related to trauma and biochemical responses. Systematic reviews, meta-analyses, in vitro model studies, case studies, and other study types were excluded.
3. Data type: Studies reporting clinical indicators of patient outcomes and trauma severity are defined by in-hospital mortality and Injury Severity Score (ISS), which provide essential data for assessing the impact of glycemic levels and cytokine responses. Patients with all types of traumatic injuries were included and considered for the analysis.
4. Time for blood glucose/cytokine measurement: Studies that quantified blood glucose, hyperglycemia, or cytokine levels (with at least IL-6 measurements) within the first 24 h of hospital admission were considered for inclusion.
5. Sufficient data on clinical outcomes (odds ratios (ORs) and their corresponding 95% confidence interval (CI)).

**Figure 3:**
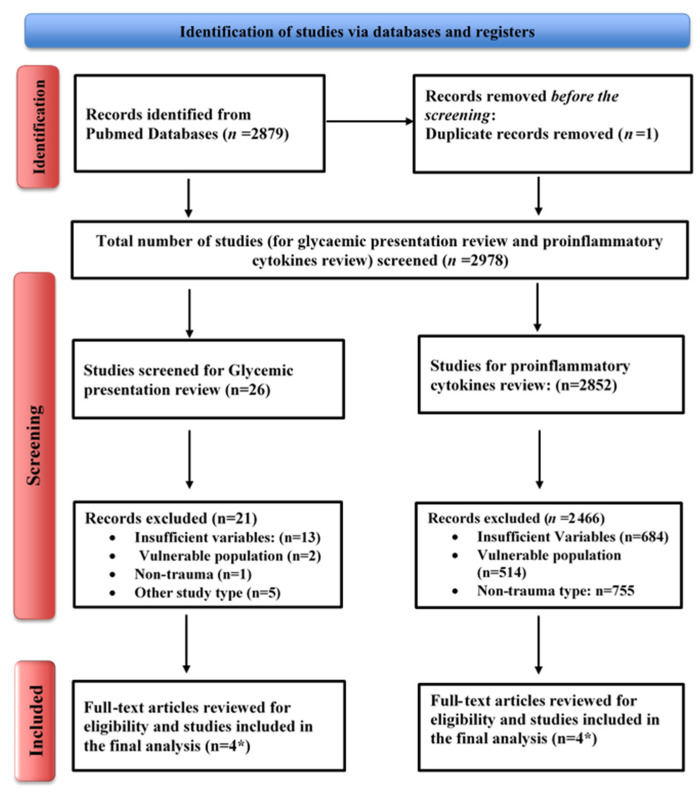
PRISMA flowchart (* one study was included in both sections).

Furthermore, only full articles in English were included. Two investigators also completed a manual search and snowballing method for additional relevant studies using references from retrieved articles. Articles were searched independently. Generally, we excluded studies if the abstract or full-text paper in English was not accessible. Studies were also excluded if they lacked sufficient information to calculate OR. The PRISMA flowchart reports detailed reasons for study exclusion [77] (Figure 3).

### 2.2. Study Selection

After the literature search, IA and NK reviewed the titles and abstracts independently. Any disagreements were resolved through mutual agreement. Studies that met the eligibility criteria were included, and those that did not were excluded with specific reasons provided. Any conflicts in study selection were discussed, and a third reviewer (AE) provided additional judgment until a consensus was achieved.

### 2.3. Data Extraction

Two investigators (IA, NK) independently conducted a full-text review of all retrieved articles. This process included data extraction and an assessment of the potential risk of bias for each study. Discrepancies arising during the data extraction stage were resolved through a consensus approach, with reviewers consulting the primary data from the original articles. Data were extracted using a customized data extraction form developed in Microsoft Excel (Redmond, WA, USA), which included information about the following variables:

- First author’s name and publication year;
- Study design (e.g., prospective or retrospective);
- The country where the study was conducted;
- Recruitment period;
- Follow-up duration;
- Characteristics of the study population;
- Sample size;
- Proportion of male/female participants;
- Participant age;
- Definition and cut-off point used for hyperglycemia diagnosis;
- Measured outcomes (including specific outcome measures such as odds ratio [OR]);
- Study conclusions.

The SIH, DH, DN, and NDN groups were analyzed for relevant outcome data, including mortality. After all reviewers agreed, studies with incomplete or inaccessible data were excluded.

### 2.4. Data Synthesis and Statistical Analysis

To address the research question, mortality was reported as the percentage of deaths within the study population. Age and gender were also considered to assess the generalizability of the population. The ISS was employed to measure injury severity, which is a standardized scoring system ranging from 0 to 75. This score is derived from the sum of the squares of the highest Abbreviated Injury Scale (AIS) values (ranging from 1 to 6) for six body systems. For the statistical analysis, point estimates of odds ratios (ORs) with their respective 95% confidence intervals (CIs) were pooled from individual studies to evaluate differences in ISS and mortality and to assess associations between different glycemic presentations, with NDN serving as the control group in trauma patients. Cytokine concentrations were compared between non-surviving and surviving patients across multiple studies. Statistical significance was tested using Pearson’s chi-squared (χ^2^) test to examine whether ISS and mortality were independent of glycemic presentations due to the variables’ large sample sizes and categorical nature. The available data on cytokines from the studies were also evaluated. As there were only a small number of studies, the graphic representation of publication bias was not performed. Analyses were performed using STATA version 14.1 software (StataCorp, College Station, TX, USA). Data were represented by 95%CI, and *p* < 0.05 was considered statistically significant.

## 3. Results

Study selection: At initial systematic searching, we retrieved a total of 2879 articles from the PubMed database using the search strategy. After excluding one duplicate article, 2878 articles were screened based on their titles and abstracts. This resulted in 2852 articles for review on proinflammatory cytokines and 26 for glycemic presentation. We further excluded 2848 articles from the proinflammatory cytokine studies due to insufficient variables (*n* = 684), non-trauma studies (*n* = 755), studies involving vulnerable populations (*n* = 513), and other reasons (*n* = 896). Similarly, from the initial set of 26 articles on glycemic presentations, 21 studies were excluded from further consideration due to reasons such as insufficient variable reporting (*n* = 13), non-trauma study design (*n* = 1), the inclusion of vulnerable populations (*n* = 2), and other study types (*n* = 5). Articles were excluded if they did not report blood glucose level parameters or outcomes of interest. Ultimately, nine articles met the inclusion criteria for systematic review, which underwent detailed data evaluation.

### 3.1. Study Characteristics

To compare glycemic presentations, we analyzed five studies [11,31,33,35,78], with the data summarized in Table 1. These studies were published between 2017 and 2021. Four articles were retrospective cohort studies, and one was a prospective cohort study. The studies included 14,750 patients, 56.29% male and 43.71% female, with a mean age of 61.4 years (Table 2).

### 3.2. Patients’ Characteristics

**Figure 4:**
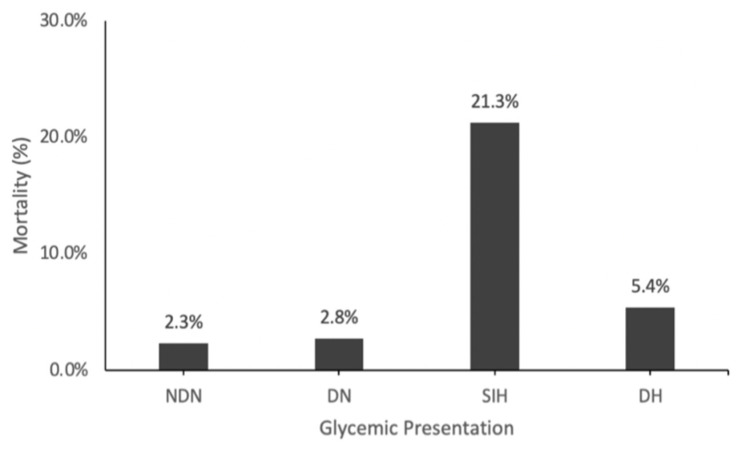
Mortality rate for glycemic presentations (based on data from prior studies).

Glycemic Presentations: Patients with SIH and NDN were predominantly male (59.3% and 59.2%, respectively), while DN and DH patients were predominantly female (57.2% and 55.9%, respectively) (*p* < 0.001) (Table 2). DN and DH patients were, on average, older (69.2 and 66.5 years, respectively) than NDN and SIH patients (53.4 and 56.5 years, respectively). Moreover, SIH patients had the most significant mortality (21.3%), followed by DH (5.4%), DN (2.8%), and NDN patients (2.3%) (*p* < 0.001) (Figure 4). Additionally, SIH patients had a higher proportion of ISS ≥ 25 (23.8%) and ISS = 16–24 (26.5%), with a lower proportion of ISS < 16 (49.6%), whereas the other glycemic groups showed higher proportions of ISS < 16 and lower proportions of ISS ≥ 25 and ISS = 16–24 (*p* < 0.001) (Table 2).

To further analyze the association between stress-induced hyperglycemia and increased mortality in trauma patients, we calculated the odds ratios (ORs) of ISS and mortality for each paired glycemic presentation to determine which glycemic group had a relatively more significant impact on ISS and mortality (Table 3).

### 3.3. SIH and Injury Severity

SIH patients were more likely than NDN patients to present with ISS = 16–24 (OR 1.78; *p* < 0.001) and ISS ≥ 25 (OR 5.80; 95% CI [4.82–6.98]; *p* < 0.001) but less likely to present with ISS < 16 (OR 0.28; *p* < 0.001). Similarly, compared to DH patients, SIH patients were more likely to present with ISS = 16–24 (OR 1.59; *p* < 0.001) and ISS ≥ 25 (OR 4.12; 95% CI [3.18–5.34]; *p* < 0.001), but less likely to have ISS < 16 (OR 0.34; *p* < 0.001). These findings suggest a potential association between SIH and more severe injuries (Table 3).

DH and Injury Severity: Interestingly, previous studies reported that DH was only associated with mortality when ISS was not controlled for. Therefore, we calculated the ORs for the association between DH and ISS to evaluate whether this relationship persisted in a large sample size (*n* = 14,750). DH patients were slightly more likely than NDN patients to present with ISS = 16–24 (OR 1.12; *p* = 0.129) and ISS ≥ 25 (OR 1.41; 95% CI [1.13–1.75]; *p* = 0.002) but slightly less likely than NDN patients to present with ISS < 16 (OR 0.82; *p* < 0.002) (Table 3).

**Figure 5:**
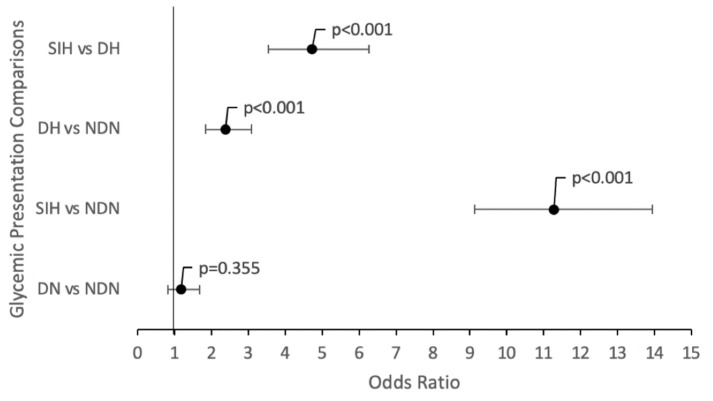
Forest plot of ORs of each paired glycemic presentation comparison in Table 3 with their respective 95% CI error bars and *p*-values.

Mortality Comparisons: SIH patients had an 11.28-fold higher likelihood of mortality than NDN patients (95% CI [9.13–13.93]; *p* < 0.001) and DH patients (OR 4.72; 95% CI [3.55–6.27]; *p* < 0.001). This was significantly higher than all other glycemic presentations (Figure 5). DH patients had slightly higher mortality odds than NDN patients (2.39; 95% CI [1.85–3.09]; *p* < 0.001), while DN patients, relative to NDN patients, failed to associate significantly with mortality (OR 1.18; 95% CI [0.83–1.69]; *p* = 0.355) (Table 3).

### 3.4. Probing the Role of Proinflammatory Cytokines in Trauma Mortality

To investigate the influence of cytokines, we utilized four studies examining the impact of TNF-α, IL-1, and IL-6 on mortality among trauma patients [35,46,47,79] (Table 4). These studies collectively underscored the association between cytokines, notably IL-6, and increased mortality following trauma. Specifically, non-survivors exhibited significantly higher levels of IL-1 and IL-6 upon admission (*p* < 0.001) compared to survivors, although TNF-α levels did not demonstrate a significant difference (*p* > 0.05) [46]. By day 2 post-admission, only IL-6 levels remained significantly elevated among non-survivors (*p* < 0.001), with no significant differences in cytokine levels observed by day 5 post-admission. This underscores the stronger association of IL-6 with mortality in trauma cases compared to IL-1 and TNF-α [46].

The conclusions drawn by Kumari et al. [46] were corroborated by a study focusing on both isolated and polytrauma cases [47], which revealed elevated IL-6 concentrations among non-survivors compared to survivors upon admission and at 6, 12, and 24 h post-admission. A significant correlation was also found between elevated IL-6 levels and 30-day mortality (*p* < 0.0001). Furthermore, Stensballe et al. emphasized the independent and significant association between IL-6 concentrations at 12 h post-admission and mortality, even after adjusting for ISS (OR 2.5; 95% CI [1.3–4.5]) [47].

Furthermore, Frink and colleagues reported that even after adjusting for injury severity using a logistic regression analysis and Receiver Operating Characteristic (ROC) analysis, IL-6 remained a significant predictor of mortality with an accuracy of 86.1% (specificity: 100%; sensitivity: 28.6%) on day 1 [79]. The area under the curve (AUC) value was calculated as 0.858 (standard error [SE]: 0.05; 95% CI [0.759–0.956]).

**Figure 6:**
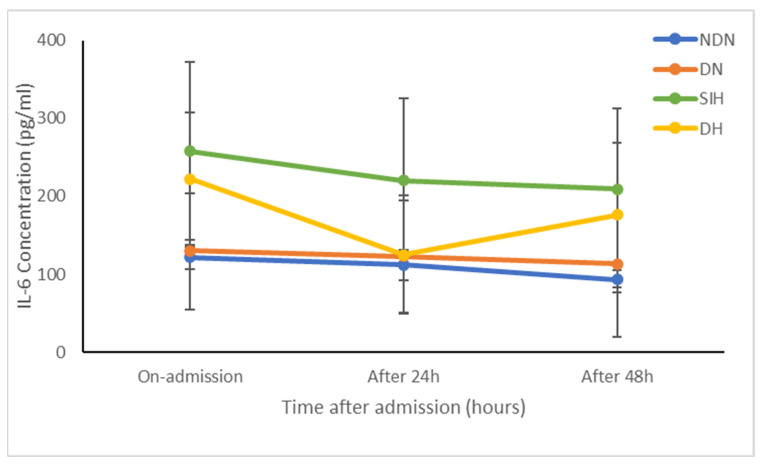
Trend in IL-6 concentration (pg/mL) on admission and at 24 and 48 h after admission across different glycemic presentations (NDN, DN, SIH, and DH) with 95% confidence interval error bars. (Data adapted from El Menyar et al., 2021 [35].

Regarding the association between IL-6 and SIH, a study focusing on polytrauma patients [35] reported that patients with SIH had elevated IL-6 concentrations compared to NDN, DN, and DH patients. These differences were statistically significant upon admission (*p* = 0.001) and at 24 h (*p* = 0.0046) and 48 h (*p* = 0.001) post-admission (Figure 6). Additionally, SIH patients exhibited the highest mortality rates, although patients with DN also experienced comparable mortality rates (*p* = 0.005).

## 4. Discussion

This structured scoping review was conducted to evaluate the impact of SIH on mortality in trauma patients, and compare it with other glycemic conditions (NDN, DN, DH), and examine cytokines’ role in these outcomes. The current systemic review found evidence to suggest that SIH is associated with more significant mortality in trauma compared to all other glycemic presentations. Furthermore, DH was also associated with mortality, showing more than a two-fold higher likelihood compared to NDN patients. This suggests that the detrimental effect of hyperglycemia on mortality extends beyond SIH. However, SIH had a significantly greater effect size, as evidenced by the 4.7-fold higher likelihood of mortality in SIH patients compared to DH patients (*p* < 0.001). Consequently, these findings suggest that both SIH and DH are associated with increased mortality in trauma cases, with SIH appearing to have a more pronounced impact.

The underlying causes of the increased mortality rates observed in trauma patients with SIH, as opposed to those with DH, are not yet fully understood. The mechanisms underlying the detrimental effects of SIH compared to DH are likely distinct. SIH is an acute process triggered by excessive adrenal cortical output (stress hormones) and cytokines, causing rapid hyperglycemia due to severe physiological stress. Conversely, DH is a chronic condition marked by persistent hyperglycemia and long-term microvascular complications [8]. These differences in pathophysiology suggest that SIH and DH impact patient outcomes, especially in trauma situations, through different biological processes.

Moreover, one explanation might be that individuals with diabetes may be better equipped to handle prolonged blood sugar levels, while non-diabetic patients may struggle more with spikes in blood sugar following severe traumatic injuries [80,81]. Notably, many research studies overlook the presence of blood sugar levels before injury in diabetic patients, and it is important to note that SIH and DH can coexist, as patients with DH may experience SIH as well [82].

Furthermore, the more significant mortality in SIH patients in the selected studies might be supported by SIH’s associated mitochondrial dysfunction and organ and cellular failure [62,83,84], which may have occurred in this study population during their hospital stay. Mitochondrial dysfunction stands as a pivotal link in the pathological processes of SIH and DH among trauma patients. Traumatic insults can cause mitochondrial dysfunction by disrupting systemic cellular functions or direct effector impacts on organelles. This dysfunction is characterized by alterations in mitochondrial numbers, deep ultrastructural abnormalities, impaired biogenesis and enzyme activity, reduced ATP synthesis, disrupted calcium homeostasis, and excessive reactive oxygen species (ROS) formation [85,86]. Moreover, oxidative stress, a key factor in SIH and DH pathology, contributes to ROS accumulation and macromolecular damage [87]. In trauma, persistent hyperglycemia intensifies ROS production, particularly within mitochondria, disrupting insulin signaling pathways [87,88]. Impaired insulin signaling exacerbates mitochondrial dysfunction, oxidative stress, and advanced glycation end-product accumulation [89]. Additionally, inflammation further aggravates mitochondrial dysfunction and vice versa, leading to an inflammatory response triggered by the release of mitochondrial components [90,91].

This review found that DN was associated with less mortality than NDN patients (OR 1.18; 95% CI [0.83–1.69]; *p* = 0.355). Other studies did not support this finding [31,35]. This anomaly must be investigated as it may be related to proinflammatory and hormonal factors. The current review found that SIH patients were more likely to have ISS scores of 16–24 and ISS ≥ 25 and less likely to have ISS < 16, compared to both NDN and DH patients (*p* < 0.001; Table 3), suggesting that severe injury may explain the more significant mortality in SIH patients and a potential association between SIH and more severe injuries. This finding aligns with the study by Cheng et al., which reported that the SIH group had significantly higher ISS scores than the NDN and DH groups [32]. However, this was countered by other studies [10,11], which reported that after adjusting for ISS, SIH, compared to NDN, was still significantly associated with mortality at a relative risk of 2.41 and OR of 3.0, respectively. Nevertheless, these values are considerably lower than the 11.28-fold increased risk of mortality observed in SIH patients compared to NDN patients in this study, indicating that ISS is still having an impact.

These studies also found that DH was not associated with increased mortality compared to NDN patients once ISS was controlled. This indicates that the mortality association is specific to SIH under these conditions. This suggests that the difference in mortality between SIH and DH patients can be attributed to metabolic differences between these conditions [11]. It is plausible that the shared hyperglycemia does not primarily drive the mortality associations, as it is confounded by injury in DH patients but not in SIH patients. However, this present review found that DH only had a slight association with the ISS ≥ 25 presentation (OR 1.41; *p* < 0.002), suggesting that this may not be the case. This needs to be investigated further.

Cytokines, such as IL-6, are strong predictors of mortality and may have an even greater impact on outcomes than SIH. Notably, a study by Kumari et al. found a more significant association between IL-6 and mortality, which was more substantial than IL-1’s and TNF-α’s associations, as IL-6 was the only cytokine with significant (*p* < 0.001) elevation in non-surviving patients compared to surviving patients on both admission and day 2 [46]. This assertion is supported by Frink and colleagues [79], who showed that elevated IL-6 remains significantly associated with mortality even after controlling ISS. However, an ROC analysis revealed a low sensitivity of 28.6% for this association, with a high false-negative rate of 71.4%, suggesting that IL-6 may be an inaccurate predictor of mortality. Nonetheless, the high AUC value (0.858) and low SE (0.05) imply some accuracy in IL-6 as a mortality predictor. As such, there is a significant association between IL-6 and mortality in trauma patients, which may be more significant than SIH’s association.

However, recent evidence, including the current analysis, counters this idea, suggesting that an interaction between IL-6 and SIH may explain the observed mortality associations. Notably, a study by El-Menyar et al. reported that SIH patients had significantly elevated IL-6 concentrations compared to all other glycemic presentations on admission, and at 24 and 48 h [35]. Therefore, the elevation of IL-6 and associated metabolic alterations are specific to SIH and are not observed in other glycemic presentations. This indicates that the combined effects of both IL-6 and SIH, rather than the independent action of a single factor, may explain their association with increased mortality. However, the sample size of the study was limited to 250 patients, and there are no similar studies to validate these findings, necessitating further investigation. Additionally, this explanation overlooks the potential impact of counter-regulatory hormones and TNF-α, which may play a significant role in this association. Despite these limitations, the findings are supported by the mechanisms underlying SIH.

Notably, for proinflammatory cascades to occur in SIH, glucose must be present to activate IKK and NFκB [19,62], causing IL-6 production [45,76,90], explaining its elevation in SIH patients. IL-6 also provokes insulin resistance and hyperglycemia by increasing CRH and ACTH release [3,19,43]. Therefore, SIH’s metabolism combined with the increase in IL-6 levels may explain some adverse effects in trauma patients that could be associated with mortality. Additionally, this could explain why SIH patients have a more significant association with mortality in trauma than DH patients. Essentially, IL-6 was more elevated in SIH trauma patients compared to DH trauma patients, suggesting that it is IL-6 that acts as a differentiating factor in the physiology of SIH and DH and the mortality associations. A recent meta-analysis concluded that SIH was associated with a higher risk of all-cause mortality in TBI patients without preexisting DM (RR: 2.00, 95% CI: 1.72–2.33) [92].

**Limitations**: The research question needs further clarity to better define traumatic injuries, as multiple categories have distinct effects on the body in terms of stress. Consequently, there may be variations in SIH metabolism. Some studies [31,33,46] focused on specific traumatic injuries and excluded severe trauma to other regions, which may have skewed the findings as SIH may have a more significant effect depending on the type and extent of the injury. The hospital length of the stay and other complications were not considered.

Another issue is that retrospective studies [11,31,33,46,78,79] comprised most of the studies included in this review. Since retrospective studies investigate the disease after it has occurred, they can only establish associations and cannot infer causality. Regarding the genders included in some studies, one study had a population of 2% females [35]. As a result, gender-biased results cannot be ignored. The mechanism of traumatic injury can vary between patients, causing variations in the outcomes. It is also possible that each person has a different tolerance and response to stressful conditions.

**Prospectives**: The role of injury severity is unclear, as SIH patients tend to have a greater risk of severe injury not seen in other glycemic presentations. Additionally, the reason why IL-6 levels were initially similar between SIH and DH patients is unclear, as these glycemic presentations have different physiologies. This, thus, needs to be investigated further. Therefore, large-scale, prospective, multicentric studies could be designed to evaluate the interdependence between SIH and IL-6 in the mortality of trauma patients, as well as to examine the differences in mortality between SIH and DH patients. Such studies would provide more robust and generalizable data, addressing the limitations of smaller sample sizes and single-center studies. Furthermore, given the association of SIH with increased mortality in trauma patients, it is imperative to investigate whether effective glycemic control could mitigate this increased risk. This research could potentially lead to improved clinical guidelines and therapeutic strategies aimed at reducing mortality in trauma patients presenting with SIH.

## 5. Conclusions

SIH is associated with a higher mortality among trauma patients, showing a significantly greater likelihood of mortality, compared to the other glycemic states and non-diabetic patients. This review also supports the interdependence between SIH and IL-6 that could explain the increased mortality associated with elevated IL-6 in SIH patients and the observed mortality differences between SIH and DH patients.

## Figures and Tables

**Table 1:** Summary studies examining glycemic presentation comparisons concerning mortality in trauma patients.

Authors	Methodology	Types of Traumatic Injuries	Number of Patients	Age (Years) Mean ± SD Median (Range)	Significant Findings
[35]	Prospective study on trauma patients (≥18 years) with random blood glucose level and HbA1c within 5 h of admission excluding pregnant women and individuals with alcohol consumption. Hyperglycemia was defined as a serum glucose level of 200 mg/dL or higher, and diabetes as an HbA1c of 6.5% or higher. The study compared four groups: NDN, DN, DH, and SIH.	Any trauma	Total = 250 NDN = 207 DN = 11 SIH = 16 DH = 16	Total = 35.1 ± 10.1 NDN = 34 (33–36) DN = 44 (36–53) SIH = 32 (28–36) DH = 41 (36–46)	Patients with SIH had the highest average ISS of 34 (*p* = 0.001), the highest mortality rate at 18.8% (*p* = 0.05), and the longest average hospital length of stay (LOS) of 26 days (*p* = 0.003) compared to patients with other glycemic conditions. DH patients had a lower mortality rate of 6.3% (*p* = 0.05). In contrast, diabetic DN patients had a similar mortality rate to SIH patients at 18.2% (*p* = 0.05).
[11]	A retrospective propensity-score-matched study on trauma patients (≥20 years) with on-admission serum glucose levels and HbA1c level or diabetes data available. Hyperglycemia was defined as ≥200 mg/dL serum glucose level. Diabetes was defined as HbA1c ≥ 6.5%. Compared NDN, DN, DH, and SIH patients.	Any trauma	Total = 10,146 NDN = 7806 DN = 950 SIH = 493 DH = 897	Total = NR NDN = 51.7 ± 19.3 DN = 67.9 ± 12.3 SIH = 56.7 ± 18.0 DH = 65.8 ± 12.4	Patients with SIH had the highest observed mortality rate at 19.5%. SIH patients also had the highest OR for mortality compared to non-NDN patients (OR 12.3; 95% CI [9.31–16.14]), and an elevated OR in a propensity-score-matched population (OR 3.0; 95% CI [1.96–4.49]). In contrast, patients with DH did not significantly correlate with increased mortality compared to NDN in the matched population (OR 1.2; 95% CI [0.99–1.38]). The study concluded that SIH, rather than DH, was linked to higher mortality after adjusting for age, sex, co-morbidities, and injury severity through propensity-score matching.
[31]	A retrospective study on trauma patients with femoral fractures (≥20 years) with AIS < 3 in other body regions, available-on-admission serum glucose level data, and HbA1c or diabetes history. No defined burn injury patients’ hyperglycemia was defined as ≥200 mg/dL serum glucose level. Diabetes was defined as HbA1c ≥ 6.5%. Compared NDN, DN, DH, and SIH patients.	Femoral fracture trauma patients with AIS < 3 in other body regions	Total = 1990 NDN = 1326 DN = 309 SIH = 75 DH = 280	Total = NR NDN = 67.1 ± 20.1 DN = 74.2 ± 10.2 SIH = 72.6 ± 13.7 DH = 72.4 ± 10.2	The study reported that patients with SIH had the highest mortality rate (4.0%) and the highest OR for mortality compared to NDN patients (OR 13.8; 95% CI [3.30–62.69]). DH is also significantly associated with increased mortality compared to NDN (OR 6.0; 95% CI [1.60–22.52]). However, SIH did not show a significantly increased risk when compared to DN (OR 1.4; 95% CI [0.37–5.26]), nor did DH (OR 0.6; 95% CI [0.20–1.89]). The study concluded that, in patients with isolated traumatic femoral fractures, both SIH and DH were associated with higher mortality compared to NDN but not compared to DN. This conclusion remained true even after adjusting for age, sex, co-morbidities, and injury severity.
[33]	A retrospective study on trauma patients (≥20 years) with moderate and severe traumatic brain injury defined as AIS ≥ 3 in the head and AIS < 3 in other body regions. Only patients with available on-admission serum glucose level data and HbA1c or diabetes history were included. Hyperglycemia was defined as ≥200 mg/dL serum glucose level. Diabetes was defined as HbA1c ≥ 6.5%. Compared SIH, DH, and NDN patients.	Traumatic brain injury	Total = 1798 NDN = 1285 DN = 186 SIH = 140 DH = 187	Total = NR NDN = 54.3+/−19.8 DN = NR SIH = 52.4+/−18.7 DH = 65.6+/−12.0	The study found that patients with SIH had the highest mortality rate at 41.4% (*p* < 0.001) and the highest OR for mortality compared to NDN patients (OR 9.1; 95% CI [6.10–13.48]). This association remained significant even in a propensity-score-matched population (OR 6.6; 95% CI [2.58–16.91]). DH did not significantly correlate with increased mortality compared to NDN in the matched population (OR 1.4; 95% CI [0.68–2.71]). The data concluded that in patients with isolated moderate to severe TBIs, SIH was linked to higher mortality compared to NDN patients, even after adjusting for age, sex, co-morbidities, intracerebral hemorrhage, and injury severity. In contrast, DH failed to exhibit the same association.
[78]	Retrospective propensity-score-matched population study on trauma patients (≥20 years) with isolated thoracoabdominal traumatic injury (AIS ≥ 3) and without polytrauma (AIS < 3 in other body regions). Only patients with available on-admission serum glucose level data and HbA1c or diabetes history were included. Hyperglycemia was defined as serum glucose level ≥ 200 mg/dL, and diabetes was defined as HbA1c ≥ 6.5%. Compared SIH, DH, and NDN patients.	Isolated thoracoabdominal injury	Total = 802 NDN = 621 DN = 50 SIH = 52 DH = 79	Total = NR NDN = 49.8+/−17.2 DN = NR SIH = 50.2+/−15.6 DH = 61.4+/−13.7	Patients with SIH had the highest mortality rate at 9.6% (*p* < 0.001), and DH patients had a similar mortality rate at 6.3% (*p* < 0.001). In propensity-score-matched populations, after adjusting for age, sex, co-morbidities, and injury severity, SIH patients continued to have the highest mortality rates compared to NDN patients (10.6% vs. 0.0%), and DH patients also had a high mortality rate compared to NDN patients (5.3% vs. 0.0%). However, the OR was similar when comparing SIH to DH patients in the matched population. The study concluded that both SIH and DH were associated with more significant mortality in trauma patients with isolated thoracoabdominal injuries compared to NDN patients.

HbA1c = glycated hemoglobin; AIS = abbreviated injury score; NDN = non-diabetic normoglycemia; DN = diabetic normoglycemia; DH = diabetic hyperglycemia; SIH = stress-induced hyperglycemia; OR = odds ratio; ISS = Injury Severity Score; TBI = traumatic brain injury.

**Table 2:** Glycemic Presentation Classification.

Variable	NDN (*n* = 11,245)	DN (*n* = 1270)	SIH (*n* = 776)	DH (*n* = 1459)	*p*-Value
**Demographics**					
Male	6658 (59.2%)	543 (42.8%)	460 (59.3%)	643 (44.1%)	
Female	4587 (40.8%)	727 (57.2%)	316 (40.7%)	816 (55.9%)	
Mean age, years	53.4	69.2	56.5	66.5	-
**ISS**					
<16	8767 (78.0%)	1111(87.5%)	385 (49.6%)	1086 (74.4%)	<0.001
16–24	1902 (16.9%)	115 (9.1%)	206 (26.5%)	270 (18.5%)	<0.001
≥25	576 (5.1%)	44 (3.5%)	185 (23.8%)	103 (7.1%)	<0.001
**Outcomes**					
Mortality	263 (2.3%)	35 (2.8%)	165 (21.3%)	79 (5.4%)	<0.001

Variables presented as mean and categorical variables as *n*, (%); χ^2^ test used for categorical variables; NDN = non-diabetic normoglycemia; DN = diabetic normoglycemia; SIH = stress-induced hyperglycemia; DH = diabetic hyperglycemia; ISS = Injury Severity Score.

**Table 3:** Odds ratio (OR) comparisons between glycemic presentations regarding injury severity and mortality.

Variables	DN vs. NDN		SIH vs. NDN		DH vs. NDN		SIH vs. DH	
	OR [95% CI]	*p*-Value	OR [95% CI]	*p*-Value	OR [95% CI]	*p*-Value	OR [95% CI]	*p*-Value
**ISS**								
<16	1.98 [1.66–2.35]	<0.001	0.28 [0.24–0.32]	<0.001	0.82 [0.73–0.93]	<0.002	0.34 [0.28–0.41]	<0.001
16–24	0.49 [0.40–0.60]	<0.001	1.78 [1.50–2.08]	<0.001	1.12 [0.96–1.28]	0.129	1.59 [1.29–1.96]	<0.001
≥25	0.66 [0.49–0.91]	0.01	5.80 [4.82–6.98]	<0.001	1.41 [1.13–1.75]	0.002	4.12 [3.18–5.34]	<0.001
**Mortality**	1.18 [0.83–1.69]	0.355	11.28 [9.13–13.93]	<0.001	2.39 [1.85–3.09]	<0.001	4.72 [3.55–6.27]	0.001

Variables presented as mean and categorical variables as *n*, (%); χ^2^ test used for categorical variables; data are expressed as OR = odds ratio; CI = confidence interval; NDN= non-diabetic normoglycemia; DN= diabetic normoglycemia; SIH = stress-induced hyperglycemia; DH = diabetic hyperglycemia; ISS = Injury Severity Score.

**Table 4:** Studies for glycemic presentation and associations with cytokine levels and mortality in trauma patients.

Author (Year)	Methodology	Significant Findings
[35]	Prospective study on trauma patients (≥18 years) with random blood glucose level and HbA1c within 5 h of admission. Pregnant women, children, or those under the influence of alcohol were excluded. Hyperglycemia was defined as ≥200 mg/dL serum glucose level. Diabetes was defined as HbA1c ≥ 6.5%. Compared NDN, DN, DH, and SIH patients. Measured levels of proinflammatory cytokines (IL-6 and IL-18).	Patients with SIH had significantly elevated IL-6 concentrations at admission and 24 and 48 h post-admission (*p* = 0.001, *p* = 0.046, and *p* = 0.001, respectively) compared to other glycemic conditions, though the IL-6 levels decreased over time in SIH patients. SIH patients also had the highest mortality rate at 18.8% (*p* = 0.005), which was similar to the mortality rate of DN patients at 18.2% (*p* = 0.005).
[79]	Polytraumatized patients (16–65 years) with ISS ≥ 16. No history of steroid use, anti-inflammatory or hormone replacement treatment, and malignancies/chronic diseases of liver, kidney, or lung(s). Measured levels of proinflammatory cytokines (IL-6, TNF-α, IL-1, IL-8, IL-18). Considered MODS, mortality, and sepsis.	The concentrations of IL-6 (r: 0.35; *p* < 0.01), IL-8 (r: 0.53; *p* < 0.01), IL-10 (r: 0.31, *p* < 0.01), and TNF-alpha (0.32; *p* < 0.01) showed correlations with the development of MODS. As a prognostic indicator for mortality on day 1 of admission, IL-6 had a specificity of 100% and a sensitivity of 28.6% (overall accuracy of 86.1%). On day 2 of admission, IL-6 had a specificity of 97.8% and a sensitivity of 19.0% (overall accuracy of 83.2%). The cut-off threshold concentration of IL-6 for mortality was 2176.0 pg/mL. The study concluded that IL-6 was a robust prognostic indicator for both mortality and MODS.
[46]	Trauma patients aged 18–65 with isolated or polytrauma blunt chest injuries were admitted to a trauma center within 24 h of injury. Patients with isolated TBIs or chemical or burn injuries, those on anti-inflammatory medications or steroid treatments, pregnant or lactating women, and individuals with other co-morbidities were excluded. Blood samples were taken to measure serum levels of various proinflammatory cytokines. The study compared patients with fatal outcomes, discharged patients, and healthy controls.	The study revealed that chest trauma patients showed significantly higher concentrations of cytokines upon admission compared to healthy controls, with notable differences observed for IL-13, IL-2, IL-6, IL-9, IL-1, TNF-α, IFN-ϓ, IL-17A, IL-17F, IL-21, and IL-22. However, when categorizing chest trauma patients based on outcomes, those with fatal outcomes exhibited notably elevated levels of IL-6, IL-17A, and IL-1 on day 1, with IL-6 remaining significantly elevated on day 2. Increased expression of proinflammatory cytokines alongside decreased expression of anti-inflammatory cytokines could lead to clinical complications in chest trauma patients, potentially resulting in fatal outcomes.
[47]	A prospective study involving 265 adult trauma patients (18 years) admitted to a trauma room. Measured IL-6 and IL-10 upon arrival and at 6, 12, and 24 h after admission.	The study found that IL-6 and IL-10 concentrations were correlated with ISS at all sampling points. Moreover, the study found that IL-6 and IL-10 concentrations were significantly higher in patients not surviving past 30 days of admission (*p* < 0.0001). The study concluded that IL-6 and IL-10 serum concentrations are correlated with injury severity and 30-day mortality following trauma.

IL = interleukin; HbA1c = glycated hemoglobin; MODS = multiple organ dysfunction syndrome; TBI = traumatic brain injury.
